# Data on detection of singlet oxygen, hydroxyl radical and organic radical in *Arabidopsis thaliana*

**DOI:** 10.1016/j.dib.2018.11.033

**Published:** 2018-11-14

**Authors:** Aditya Kumar, Ankush Prasad, Michaela Sedlářová, Pavel Pospíšil

**Affiliations:** a Department of Biophysics, Centre of the Region Haná for Biotechnological and Agricultural Research, Faculty of Science, Palacký University, Šlechtitelů 27, 783 71 Olomouc, Czech Republic; b Department of Botany, Faculty of Science, Palacký University, Šlechtitelů 27, 783 71 Olomouc, Czech Republic

**Keywords:** Photosystem II, Reactive oxygen species, Hydroxyl radical, Singlet oxygen, Organic radical

## Abstract

This article contains data related to the research article entitled, “Organic radical imaging in plants: Focus on protein radicals” (Kumar et al., 2018). The data presented herein focus on reactive oxygen species (ROS) and organic radical formed within photosynthetic tissues of *Arabidopsis thaliana* during high light stress and includes (1) Confocal laser scanning microscopic images using 3′-p-(hydroxyphenyl) fluorescein (HPF) as specific probe for the detection of hydroxyl radical (HO^•^); (2) Confocal laser scanning microscopic images using Singlet Oxygen Sensor Green (SOSG) as a specific probe for the detection of singlet oxygen (^1^O_2_) and; (3) Electron paramagnetic resonance (EPR) spectroscopy using spin traps for the detection of organic radical.

**Specifications table**

**Table:** 

Subject area	Biology
More specific subject area	Redox Biology
Type of data	Images/figures
How data were acquired	Fluorescence of specific fluorochromes was localized within *Arabidopsis* leaves kept in dark or illuminated with high red light (RL, λ ≥ 600 nm) by confocal laser scanning microscopy (fluorview 1000 unit attached to IX80 microscope; Olympus Czech Group, Prague, Czech Republic) and electron paramagnetic resonance (EPR) spectra were taken in thylakoid membranes isolated from *Arabidopsis* leaves illuminated with high white light (WL) using EPR spectrometer MiniScope MS400 (Magnettech GmbH, Berlin, Germany).
Data format	Analyzed
Experimental factors	Leaves and thylakoid membranes of *Arabidopsis* plant were used.
Experimental features	1. Plants were pre-illuminated with high white light (1500 µmol photons m^−2^ s^−1^) at low temperature (8 °C) for 13 h to induce oxidative stress. 2. Leaf tissues were infiltrated with fluorescent probes, 3′-p-(hydroxyphenyl) fluorescein (HPF) or singlet oxygen sensor green (SOSG), and incubated for 30 min at room temperature in dark (control) or illuminated with RL. Fluorescence was visualized subsequently by confocal laser scanning microscopy. 3. Thylakoid membranes were isolated from pre-illuminated leaves and illuminated by a high white light for 30 min/5 min (as specified). Formation of organic radicals was determined using EPR spin trapping spectroscopy.
Data source location	Department of Biophysics, Centre of the Region Haná for Biotechnological and Agricultural Research and Department of Botany, Palacký University, Olomouc, Czech Republic. Loc: 49°34′33.828″N, 17°16′54.658″E
Data accessibility	The data are available within this article
Related research article	Aditya Kumar, Ankush Prasad, Michaela Sedlářová, Pavel Pospíšil, Organic radical imaging in plants: Focus on protein radicals (in press, Free Radical Biology and Medicine) doi.org/10.1016/j.freeradbiomed.2018.10.428[1].

**Value of the data**

• We suggest organic radicals and other biomolecules oxidized by HO^•^ and ^1^O_2_ as significant members of the signaling pathways in high-light stressed plants.
• We illustrate the localization of hydroxyl radical (HO^•^) and singlet oxygen (^1^O_2_) within *Arabidopsis* leaves as well as detection of organic radicals in thylakoid membranes by techniques which might be of interest to the community of plant redox biology and ROS-mediated signaling.
• Apart from malondialdehyde (MDA), organic radicals/ biomolecules oxidized by HO^•^ and ^1^O_2_ have not been reported as major regulators of signaling in stressed plants. However, our current data article can be useful as it deals with the current aspect and hypothesizes the phenomenon.
• We present histochemical staining followed by confocal laser scanning microscopy which can be used as an imaging tool for HO^•^, ^1^O_2_ localization and spin-trap based EPR spectroscopy for qualitative analysis of organic radical.

## 1 Data

Data presented herein bring together microscopic techniques used to visualize the formation of ROS (HO^•^ and ^1^O_2_) within *Arabidopsis* leaves in the first stage and detection of organic radical formation in the second stage of stress-induced protein oxidation as suggested in [1]. Plants were subjected to high white light stress (1500 µmol photons m^−2^ s^−1^) at low temperature (8 °C) for 13 h, i.e. conditions chosen so as to reach oxidative stress in photosystem II but concurrently to avoid chlorophyll degradation. Immediately after the light-induced stress, the leaves were cut into pieces and incubated with specific fluorescent probes in dark/RL for 30 min to gain confocal images from spongy mesophyll cells, omitting 3 lines of cells along the injured edges. To avoid photosensitization of SOSG [2], an RL source (*λ* ≥ 600 nm) has been used during histochemical staining, recently with both fluorescent probes. Organic radicals formation were determined using EPR spin trapping spectroscopy in thylakoid membranes isolated from pre-illuminated *Arabidopsis* leaves and illuminated by a high white light (30 min/5 min, as specified).

## 2 Experimental design, materials and methods

### 2.1 Plant samples – *Arabidopsis thaliana*

Seeds of *A. thaliana* wild-type Columbia-0 was purchased from the Nottingham *Arabidopsis* Stock Centre (NASC), U.K. The seeds were soaked for 4 days (at 4 °C) followed by potting with a peat substrate (Klasmann, Potgrond H). The plants were grown for 5–6 weeks in a walk-in type growth chamber Fytoscope FS-WI-HY (Photon Systems Instruments, Drásov, Czech Republic) under following conditions: photoperiod of 8/16 h light/dark (100 μmol photons m^−2^ s^−1^); temperature of 22/20 °C light/dark and a relative humidity of 60%. For high light treatment, illumination at 1500 µmol photons m^−2^ s^−1^ at low temperature (8 °C) for 13 h was achieved using AlgaeTron AG 230 (Photon Systems Instruments, Drásov, Czech Republic).

### 2.2 Fluorescent probes and spin traps

3′-p-(hydroxyphenyl) fluorescein (HPF) (ThermoFisher Scientific, Paisley, UK) and SOSG (Molecular Probes Inc., Eugene, OR, USA) were infiltrated to leaf tissues for confocal laser scanning microscopy in order to detect and localize HO^•^ and ^1^O_2_, respectively. For EPR spin trapping spectroscopy, α-(4-pyridyl N-oxide)-N-tert-butyl nitrone (POBN) (Sigma Aldrich, GmbH, Germany) and 5,5-dimethyl-1-pyrroline N-oxide (DMPO) (Dojindo Molecular Technologies Inc. Rockville, MD, USA) were used to detect organic radicals in thylakoids.

### 2.3 Histochemical staining for microscopy

Leaf pieces of size 5 × 5 mm were excised from *Arabidopsis* leaf blade on a glass slide wetted with HEPES buffer (pH 7.5) and infiltrated with fluorescent probes (either 10 μM HPF or 50 μM SOSG) in a syringe. Subsequently, the leaf pieces together with probe solution were transferred into 1.5 ml Eppendorf tube and incubated for 30 min either in dark or exposed to high red light. The illumination was performed utilizing a LED source with a light guide CL6000 LED Zeiss (Carl Zeiss Microscopy GmbH, Jena, Germany). The exposure was achieved using a long-pass edge interference filter (*λ* ≥ 600 nm) (Andover Corporation, Salem, NH, USA). Fig. 2A shows a schematic representation of the experimental procedure for details refer to references [2], [3].

### 2.4 Confocal laser scanning microscopy

**Fig. 1:**
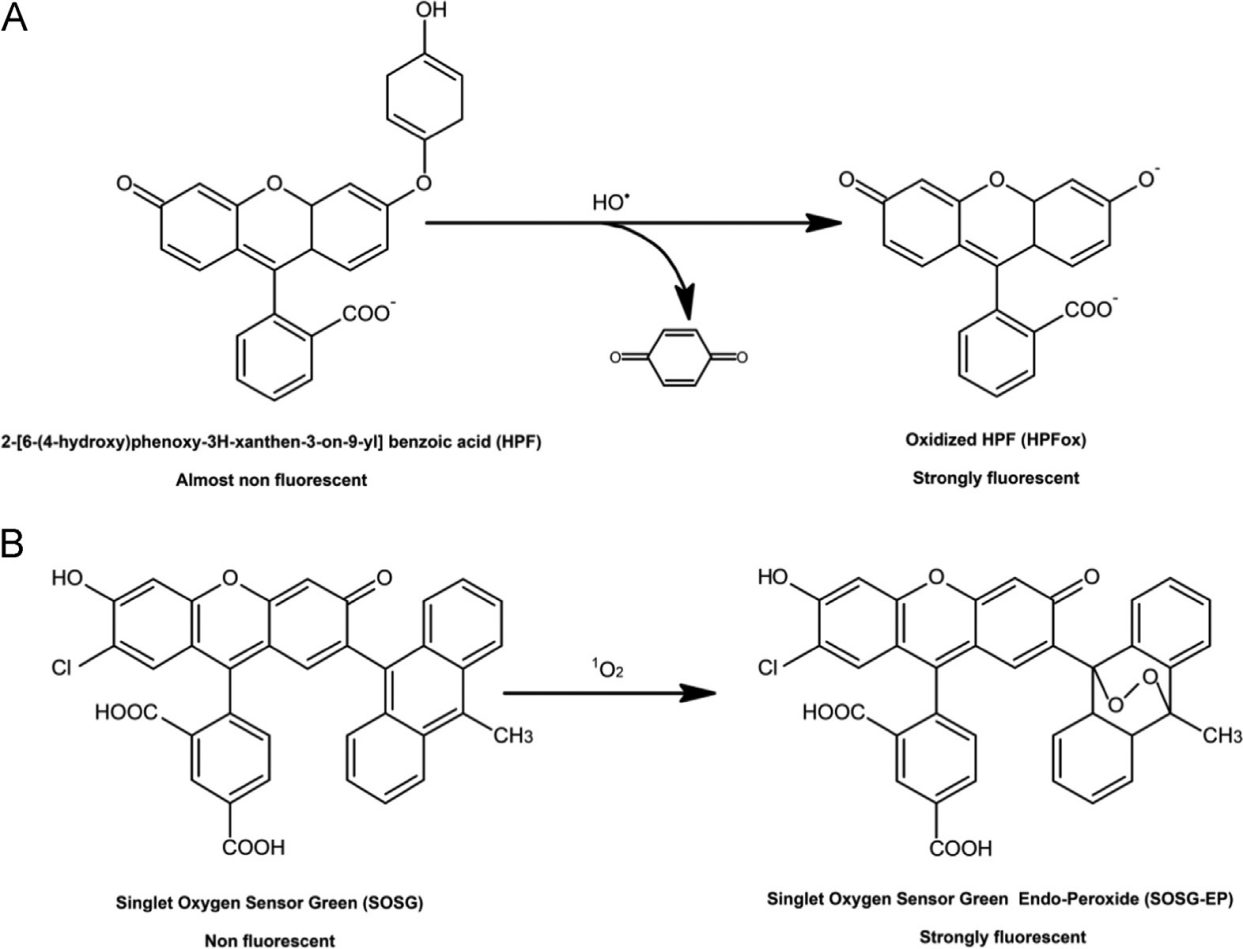
(A) Non-fluorescent hydroxyphenyl fluorescein (HPF) oxidized by hydroxyl radical (HO^•^) form a highly fluorescent compound HPF-ox which exhibits bright green fluorescence (excitation/ emission maxima ~490/515 nm). (B) Singlet oxygen sensor green (SOSG) oxidized by singlet oxygen (^1^O_2_) forms SOSG endoperoxide (SOSG-EP) providing bright green fluorescence (excitation/emission maxima ~504/525 nm) (as per datasheet, ThermoFisher Scientific, Paisley, UK and Molecular Probes Inc., Eugene, OR, USA).

**Fig. 2:**
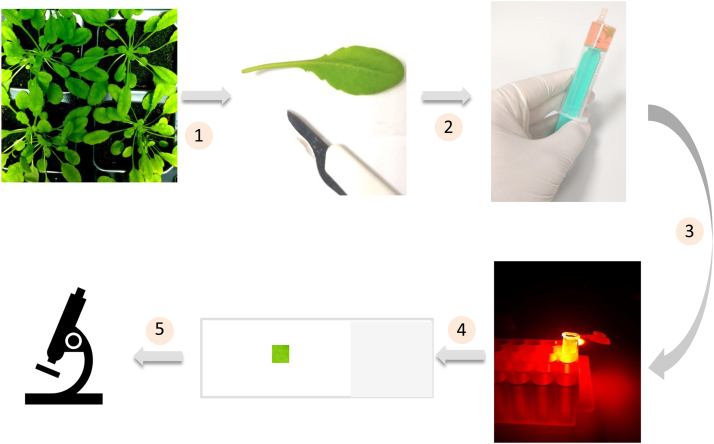
A schematic representation showing the different steps involved in the preparation of samples for confocal laser scanning microscopy.

**Fig. 3:**
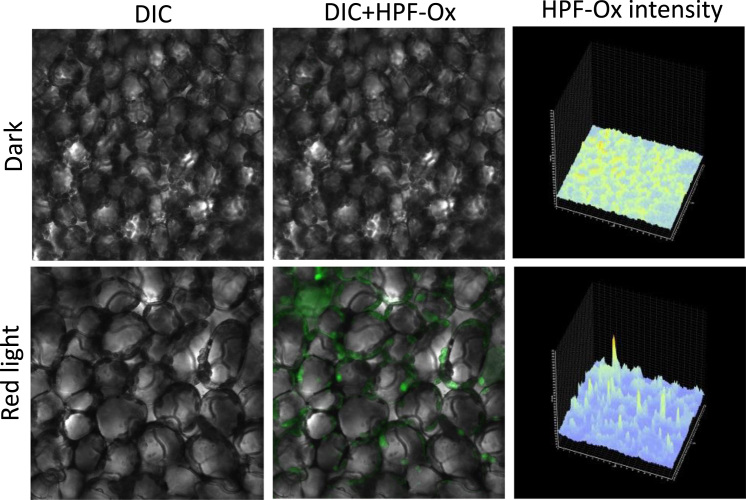
Hydroxyl radical imaging in *Arabidopsis* leaves. *Arabidopsis* leaves were infiltrated with 10 μM HPF in dark (upper panel) or exposed to high red light (lower panel) for 30 min. From left to right is Nomarski DIC channel, combination of Nomarski DIC channel+HPF-ox fluorescence and integral distribution of the HPF-ox fluorescence signal intensity within the sample.

**Fig. 4:**
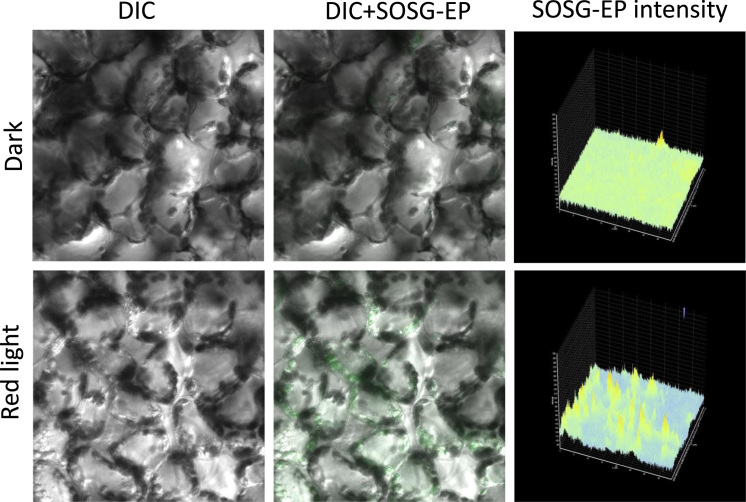
Singlet oxygen imaging in *Arabidopsis* leaves. *Arabidopsis* leaf tissues were infiltrated with 50 μM SOSG in dark (upper panel) or exposed to high red light (lower panel) for 30 min. From left to right are Nomarski DIC channel, combination of DIC+ SOSG-EP fluorescence channels and integral distribution of the SOSG-EP fluorescence signal intensity within the sample.

Following the staining procedure, the leaf pieces were transferred into a fresh HEPES buffer (pH 7.5) on a slide and visualized by confocal laser scanning microscopy (Fluorview 1000 unit attached to IX80 microscope; Olympus Czech Group, Prague, Czech Republic). The excitation of HPF and SOSG was performed by a 488 nm line of an argon laser and the emission was detected by a 505–525 nm filter, respectively. The excitation/emission parameters utilized is slightly different as compared to excitation/emission maxima mentioned in datasheet (please refer to legend to Fig. 1) due to variation of the instrument used in our study. The laser intensities for Fig. 2, Fig. 3, Fig. 4 were set as described in our previous study [2].

### 2.5 Thylakoid membrane isolation

Thylakoid membranes were prepared according to the protocol of Casazza and co-workers [4] as briefly described below.

• Leaves were harvested from 5–6 weeks old *Arabidopsis* plants.
• The leaves (5–10 g) were floated in dark on ice cold water for 5–10 min and then blotted. All glassware׳s were pre-cooled before use and all further steps were performed at 4 °C under dark condition.
• Leaves were promptly homogenized in grinding buffer (100–200 ml) containing EDTA (5 mM), EGTA (5 mM), MgCl_2_ (5 mM), sorbitol (0.4 M), NaHCO_3_ (10 mM), Tricine/NaOH (20 mM, pH 8.4), 0.5% (w/v) fatty acid-free BSA.
• The homogenate was then filtered through 2 layers of cheesecloth (moderate hand pressure) to increase the final yield of thylakoid membranes.
• The filtrate was centrifuged at 2600*g* (3 min).
• The pellet was re-dissolved in re-suspension buffer [sorbitol (0.3 M), EDTA (2.5 mM), MgCl_2_ (5 mM), NaHCO_3_ (10 mM), HEPES (20 mM, pH 7.6), 0.5% (w/v) fatty acid-free BSA].
• Suspension was centrifuged at 2600*g* (3 min, 4 °C); the pellet was washed again in re-suspension buffer and then was suspended in 50–100 ml of hypotonic buffer [EDTA (2.5 mM), MgCl_2_ (5 mM), NaHCO_3_ (10 mM), HEPES (20 mM, pH 7.6), 0.5% (w/v) fatty acid-free BSA] followed by a last-step centrifugation (2600*g*, 3 min at 4 °C).
• The pellet was dissolved in a small volume (0.5–1 ml) of the re-suspension buffer; chlorophyll concentration was calculated from the absorbance of an 80% (v/v) acetone extract measured at 645 and 663 nm [5].

### 2.6 EPR spin-trapping spectroscopy

**Fig. 5:**
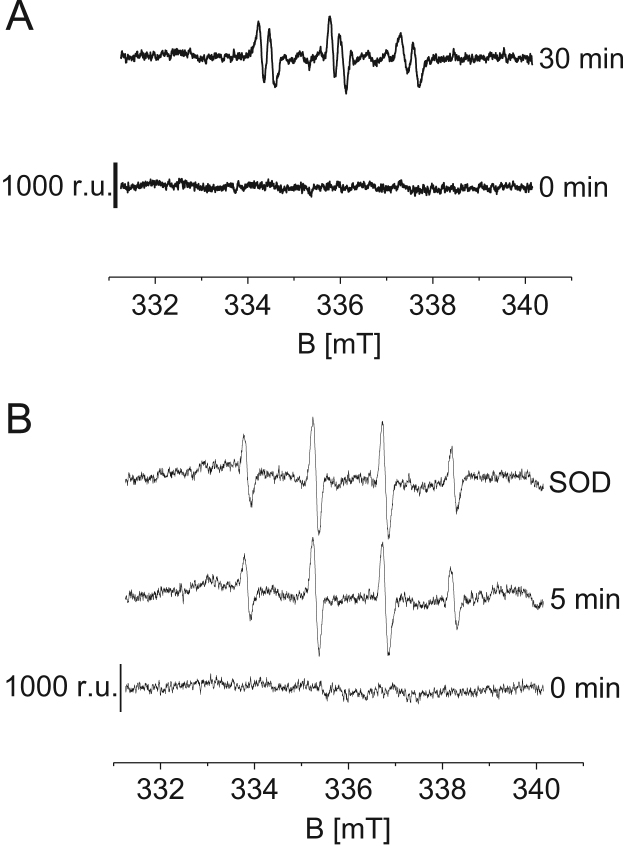
Detection of organic radical by using EPR spin trapping spectroscopy thylakoid membranes. (A) Thylakoid membranes (200 μg Chl ml^−1^) were illuminated with high white light in the presence of 50 mM POBN at 0 min and 30 min. (B) Thylakoid membranes (200 μg Chl ml^−1^) were illuminated with high white light in the presence of 50 mM DMPO at 0 min (lower trace) and 5 min [in absence (middle trace) and presence (upper trace) of superoxide dismutase (SOD) (400 U/ml)].

Organic radicals including alkyl (R^•^) and peroxyl/alkoxyl (ROO^•^/RO^•^) radicals formed in thylakoid membranes (200 μg Chl ml^−1^) were detected using EPR spin trapping spectroscopy (Fig. 5). Detection of R^•^ was performed using spin trap POBN (50 mM) while ROO^•^/RO^•^ was detected using spin trap DMPO (50 mM). High white light illumination (1500 µmol photons m^−2^ s^−1^, 30 min) was achieved using a LED source with a light guide CL6000 LED Zeiss (Carl Zeiss Microscopy GmbH, Jena, Germany). POBN-R and DMPO-OOR/DMPO-OR adduct EPR signal spectra were recorded under EPR conditions as follows: microwave power (10 mW), modulation amplitude (1 G), modulation frequency (100 kHz), sweep width (100 G), scan rate (1.62 G s^−1^).
