# Supplementary material for: Data on detection of singlet oxygen, hydroxyl radical and organic radical in Arabidopsis thaliana
Source: Data Brief. 2018 Nov 14;21:2246–52. doi: 10.1016/j.dib.2018.11.033 (PMC6276547; doi:10.1016/j.dib.2018.11.033)
Supplement: Supplementary file 1 — Supplementary material. [file mmc1.docx]

**Conflict of Interest Statement**

The authors declare that the research was conducted in the absence of any commercial or financial relationships that could be construed as a potential conflict of interest.

On behalf of all authors,

Pavel Pospíšil

Associate Professor, Department of Biophysics

Faculty of Science, Palacký University

Šlechtitelů 27, 783 71 Olomouc

Czech Republic

Tel: +420 58 5634174

E-mail: pavel.pospisil@upol.cz

http://biofyzika.upol.cz/en
